# The Mortuary Unit. Some Preliminary Considerations—I

**Published:** 1912-08-17

**Authors:** 


### THE MAKING OF A MODERN HOSPITAL.

### The Mortuary Unit.

### Some Preliminary Considerations—I.

One of the most important units or stations of the modern hospital is the mortuary. It is, alas, also one of the units which is often neglected in a manner that calls for the severest censure. Those who visit a modern hospital, and who wish to form some idea of the manner in which the administration fulfil their obligations towards the patients and towards the community, cannot do better than inspect the mortuary attached to the institution. That unit is, in a sense, a test, and a good one, of the way in which the administrators are sensible of the fact that in a modern hospital the most humane principles rule. We have progressed considerably since the days when the hospital was a dead-house and a home for the dying. We have in a measure dispelled the fear of the public that the hospital is something to be avoided because of the horrors which were extravagantly and erroneously associated with it. We have reformed public sentiment with regard to such institutions, but we have yet to do a great deal before we can claim that our hospitals need no longer fear inspection by humanitarians who maintain that the claims of the dead have to be considered just as much as those of the living.

### Disregard of Essentials in Modern Hospitals.

Most of our readers know in what state some of the mortuaries attached to our large hospitals, provincial and metropolitan, are to-day; how intolerably bad some of these units are, and how flagrantly they outrage sentiment and principles of decency. The many institutions in which these units are excellent are in the minority, and they will continue to remain in the minority until lay committees and hospital workers take an active interest in the mortuary, and recognise what are the ideal lines upon which these units should be managed, constructed, and kept up.

In this preliminary article we shall briefly outline those general principles which should guide the hospital architect in designing the mortuary block, and shall illustrate them by plans and photographs of some modern mortuary blocks. But before doing so it is advisable to consider somewhat more fully than is usually done the principal points which should be attended to in discussing the efficiency of the mortuary block in general. When we take up recent works on hospital construction, we find that as a rule the mortuary block is treated with scant consideration. So excellent and well written a manual as Dr. Mackintosh's does not devote even a paragraph to the mortuary unit; the German standard work by Ruppel deals with this unit in a cursory and non-committal fashion, which is in strong contrast to the admirable manner in which other points of construction are handled. In the journals devoted to hospital construction and architecture there is a remarkable want of papers dealing with mortuaries. We know of no article, for instance, which describes in detail the ideal mortuary, and we are acquainted with only a few books in which the subject is dealt with in a lucid and informative manner. Of these latter the best description of the modern mortuary is that given by Depage and van der Velde in their monumental work on hospital construction in Belgium, a work which is inaccessible to the lay reader,* and which so bristles with technicalities that the ordinary committeeman, if he is neither architect nor builder, will find it difficult to understand.

### The Lack of Humanitarian Principles.

What is the reason of this strange silence on mortuaries? We draw no invidious distinction when we state that in England the humanitarian aspect of hospitals has always been kept in the foreground while abroad it is more the scientific and educational aspects that have attracted attention. Only of late years have Continental authorities realised that the mortuary block of a hospital should be open to public inspection just as much as any other part of the institution. The advantages of public inspection, and of publicity in general, in everything that concerns a hospital need scarcely be commented upon. There are, and should be, no secrets in a hospital; every unit and department must be prepared to declare its work, and to show to the public that it labours for the good of the community primarily and not for the advancement of medical science, the education of doctors, or the trial of new methods. These latter are secondary considerations, however important or lost sight of by ignorant publicists, but they are not the primary considerations. The first essential of a hospital is that it should treat the sick, and that it should regard its patients as human beings who have human sentiments, feelings, and desires, which should be respected, not ignored.

### The Primary Necessities.

What does this involve in the case of the mortuary block? This, firstly, that the mortuary must be regarded as the sanctuary of the institution, the place where the dead can rest in peace and calm, where their friends can take a last look at them undisturbed by the activity and bustle which are ordinary accompaniments of hospital life. Where the dead patient is treated solely as a "subject" for anatomical purposes, where the *post-mortem* room is the first consideration of the architect who designs the mortuary, and where the annexes, the mortuary chapel, the visitors' room, and the undertaker's rooms are regarded as essentially secondary matters, it is impossible that the mortuary block can be ideal. In Continental hospitals, where dozens of *post-mortem* examinations take place daily, where the patients are virtually infirmary patients, and where nearly everything is made subservient to pathological research, it is not to be wondered at that the principles of mortuary construction have been disregarded in the past. In justice to modern

* It may be seen at The Hospital Library, 28 Southampton Street, Strand.

hospital constructors on the Continent, it must, however, be admitted that during recent years a great change has come over the attitude of hospital constructors on this question of mortuaries. It is becoming recognised that the mortuary must fulfil certain conditions which common decency, religious sentiment, and respect for the dead demand. We shall have occasion to refer to some recent institutions in which these conditions have been met in a manner that is almost exemplary and well worthy of study by hospital workers in this country.

### How to Obtain Them.

It may be asked: How is it possible to ensure these conditions and to make the mortuary a place of quiet, calm privacy when one of its most important features is the *post-mortem* room, admittedly a locale for the dissection of dead bodies? To the average patient and to his friends the thought of *post-mortem* dissection is revolting in the extreme. We, who know how these after-death examinations are carried out, and who realise how indispensable they are in the vast majority of cases, must admit that the public has grave objections to *post-mortem* dissections. It is our duty to educate the public to realise, as we already realise, that they are necessary and involve no desecration or contumely. The public prejudice against dissection after death has arisen, largely if not wholly, from the unfortunate proviso in old laws that gave the murderer's body to the medical school for public anatomising. The repulsion engendered by this legal enactment, now repealed, still lingers, though it is undoubtedly dying out, as the public is becoming aware of the fact that medical knowledge is advanced by the information gained by *post-mortem* examinations. But the feeling will remain so long as hospitals continue to have mortuaries which are a disgrace to the institutions that own them; places into which no layman can be brought to witness the manner in which the dead are disposed of without creating in him a disgust and a feeling of nausea. Even with a *post-mortem* room attached there is no reason why a hospital mortuary should not be one of the most attractive units of the hospital. And it can be made such if only superintendent and architect will combine to plan the block in accordance with the ideal which is ever before the humanitarian hospital worker.

### Designing the Unit.

In discussing the question it is well to bear one or two salient points clearly in mind. There are different interests to be considered in planning the block. Let us see what these are. In the first place there are the interests of the dead patient and his friends. We put these first, for they must always be paramount, no matter how enormous are the subsidiary interests of science or of the institution. Only in one instance do they lapse, and that is when the law steps in and, in the interests of the general community, demands a legal examination of the dead body. But ordinarily, hospital workers would do well to bear in mind that hospital authorities have no right to a body; no matter whether the rules of the institution prescribe that a *post-mortem* examination should be held, such examination can take place only if the dead patient's relatives and friends have consented to allow it to be performed. The law on this point is quite clear, and hospitals respect it by duly informing friends and relatives of the patient's death and asking for leave to make a *post-mortem* as a matter of routine. In very few instances, indeed, where a *post-mortem* examination is necessary, will it be found impossible to obtain consent. A tactful house surgeon or house physician will always succeed in explaining the necessity of the case and convincing the friends that the examination involves no desecration.

### Institutional Interests.

Secondly, there are the interests of the institution. It is admittedly desirable that every patient who has died in a hospital should be examined after death; only in that way, in the majority of cases, can certain diagnosis of the cause of death be obtained. It may be argued that if a patient has died of typhoid fever, a disease which is well marked in life and of which the pathological conditions found after death are well known, it is a work of supererogation to dissect the body after death. But the argument does not hold in full, for there is always information to be gleaned by *post-mortem* examination, if only in establishing the extent of the lesions which have been guessed at during life. Very often, too, such examination is a corrective of diagnosis made during life, and reveals conditions which have been previously unsuspected or have escaped recognition. It cannot be said that the failure to diagnose the case at the bedside is a sign of want of skill on the part of the medical attendant. The public, as a rule, judges that the demand to be allowed to perform a *post-mortem* examination is, in fact, an admission of ignorance on the part of the doctor. That is true in a certain sense. So long as our knowledge of pathological conditions is incomplete, we must remain ignorant of a great number of lesions and conditions which produce them. Knowledge and information can only be gathered by routine examinations, and in the interests of the patients themselves, as in those of the institution and of medical science in general, it is imperative that every modern hospital should have a *post-mortem* room.

### The Mortuary Attendant.

Thirdly, there are the interests of the workers. These, like the interests of the patient's friends, are often overlooked. The mortuary attendant is one of the most hardworked individuals on the hospital staff; his work is carried on under conditions which, to say the least, are most trying. Very often he lives in an atmosphere which is strongly reminiscent of a charnel-house; the lavatory accommodation provided for his use is of the most meagre description. Similarly, the doctors and students who have to work with him are compelled to work in a similar atmosphere under similar conditions. To all these points the architect or whoever is responsible for the planning of the mortuary block must give due consideration. Only by keeping in view all the different interests involved, and by designing the hospital mortuary in such a way that these interests may be safeguarded, can the final plan be ideal.

